# Clinical diagnosis of partial or complete anterior cruciate ligament tears using patients' history elements and physical examination tests

**DOI:** 10.1371/journal.pone.0198797

**Published:** 2018-06-12

**Authors:** Simon Décary, Michel Fallaha, Sylvain Belzile, Johanne Martel-Pelletier, Jean-Pierre Pelletier, Debbie Feldman, Marie-Pierre Sylvestre, Pascal-André Vendittoli, François Desmeules

**Affiliations:** 1 School of Rehabilitation, Faculty of Medicine, University of Montreal, Montreal, Quebec, Canada; 2 Orthopaedic Clinical Research Unit, Maisonneuve-Rosemont Hospital Research Center, Montreal, Quebec, Canada; 3 Department of Surgery, Maisonneuve-Rosemont Hospital, Montreal, Quebec, Canada; 4 Department of Surgery, Laval University Hospital Center (CHUL), Laval University, Quebec, Quebec, Canada; 5 Osteoarthritis Research Unit, University of Montreal Hospital Research Center (CRCHUM), Montreal, Quebec, Canada; 6 University of Montreal Hospital Research Center (CRCHUM), Montreal, Quebec, Canada; Copenhagen University Hospital, DENMARK

## Abstract

**Objective:**

To assess the diagnostic validity of clusters combining history elements and physical examination tests to diagnose partial or complete anterior cruciate ligament (ACL) tears.

**Design:**

Prospective diagnostic study.

**Settings:**

Orthopaedic clinics (n = 2), family medicine clinics (n = 2) and community-dwelling.

**Participants:**

Consecutive patients with a knee complaint (n = 279) and consulting one of the participating orthopaedic surgeons (n = 3) or sport medicine physicians (n = 2).

**Interventions:**

Not applicable.

**Main outcome measures:**

History elements and physical examination tests performed independently were compared to the reference standard: an expert physicians’ composite diagnosis including history elements, physical tests and confirmatory magnetic resonance imaging. Penalized logistic regression (LASSO) was used to identify history elements and physical examination tests associated with the diagnosis of ACL tear and recursive partitioning was used to develop diagnostic clusters. Diagnostic accuracy measures including sensitivity (Se), specificity (Sp), predictive values and positive and negative likelihood ratios (LR+/-) with associated 95% confidence intervals (CI) were calculated.

**Results:**

Forty-three individuals received a diagnosis of partial or complete ACL tear (15.4% of total cohort). The Lachman test alone was able to diagnose partial or complete ACL tears (LR+: 38.4; 95%CI: 16.0–92.5). Combining a history of trauma during a pivot with a “popping” sensation also reached a high diagnostic validity for partial or complete tears (LR+: 9.8; 95%CI: 5.6–17.3). Combining a history of trauma during a pivot, immediate effusion after trauma and a positive Lachman test was able to identify individuals with a complete ACL tear (LR+: 17.5; 95%CI: 9.8–31.5). Finally, combining a negative history of pivot or a negative popping sensation during trauma with a negative Lachman or pivot shift test was able to exclude both partial or complete ACL tears (LR-: 0.08; 95%CI: 0.03–0.24).

**Conclusion:**

Diagnostic clusters combining history elements and physical examination tests can support the differential diagnosis of ACL tears compared to various knee disorders.

## Introduction

The anterior cruciate ligament (ACL) is a major stabilizing structure of the knee against excessive anterior translation and internal rotation of the tibia [1, 2]. An ACL tear, either partial or complete, is a significant knee injury. The overall incidence rate in the general population varies between 30 to 80 per 100000 persons-years [3–5]. This injury is particularly common in younger individuals as it occurs approximately 70% of the time as a result of a trauma in a sporting activity requiring pivot such as football, soccer, basketball or alpine skiing [1, 5–7]. In the United States, this translate to about 250 000 ACL tears annually [7]. Of these individuals, 50 to 75% will opt for an ACL reconstruction surgery which incurs costs of up to two billion dollars [1, 3, 5, 8].

The clinical profile of this injury has serious implications for the health of individuals as it can lead to sustained complaints of instability in daily and sporting activities as well as subsequent risk of meniscal tear or chondral damage [1, 4–6, 9, 10]. To reduce disabilities in this population, an early diagnosis is advocated to guide toward accelerated rehabilitation protocols or surgical management [1, 4–6, 9, 10].

As ACL tears represent only 4% of all knee disorders in primary care settings and 15% in secondary care settings, efficiently identifying ACL tears in the clinic compared to diverse knee disorders is often challenging [11, 12]. Indeed, evidence shows that only 10 to 15% of patients with an ACL tear are correctly diagnosed upon initial presentation and too often is based on unnecessary imaging tests [10, 13, 14]. Patients have to consult multiples health providers before a valid diagnosis is established, which delays rehabilitation or surgical management [10, 13, 14]. These concerning statistics reinforce the need to validate the clinical diagnosis based on history and physical examination tests for ACL tears [10].

Diagnostic validity of physical examination tests for ACL tears has been extensively studied [15]. The three most studied tests are the Lachman, the pivot shift and the anterior drawer test [15]. These three tests reach high specificity and can be used alone to make a valid ACL diagnosis, but only the Lachman reached moderate sensitivity [15, 16]. Combining multiple history elements and physical examination tests is advocated to increase the validity of the diagnosis of ACL tears. History elements such as a pivoting traumatic event, a “popping sensation” or immediate effusion following trauma can be useful to clinicians to make a valid ACL diagnosis, yet limited evidence is available to support this approach [11].

In the hand of an expert evaluator a clinical examination for ACL tears can be equivalent to magnetic resonance imaging (MRI) findings [11]. Specific combinations of history elements and/or physical examination tests have been reported in a few studies, but results are often incompletely presented and difficult to interpret [11, 16–21]. The studies reporting more complete results, useful to clinicians in their assessment of patients with suspected ACL tears [22, 23], have in turn several methodological limitations [24], including the presence of spectrum bias [23], inconsecutive sampling [23], use of MRI only as the reference standard [22] and small sample sizes [22, 23].

Therefore, the aim of this diagnostic study is to assess the diagnostic validity of combining an extensive set of selected history elements and physical examination tests to diagnose or exclude partial or complete ACL tears compared to other common knee disorders based on a composite reference standard using standardized physical assessment and imaging.

## Methods

### Study design and settings

This is a prospective multi-center diagnostic study aimed at developing a series of diagnostic clusters for various common knee disorders. The present paper reports on results specific to ACL tears. We recruited consecutive new patients consulting one of the participating physicians for a knee complaint between November 2014 and August 2016 in two outpatient orthopaedic clinics and two primary care family medicine clinics from two urban centers in the province of Quebec in Canada. We also invited via email persons from a university community (academic staff, students or administration personnel) who had a knee complaint and needed to see a health practitioner for their knee problem.

The present study, its design, methodology and reporting of results conform to the Standards for Reporting Diagnostic Accuracy Studies 2015 (STARD) [25, 26]. The study was approved by Hospital Maisonneuve-Rosemont ethics committees and all participants signed an informed consent form.

### Participants

Potential participants were initially screened by telephone to assess preliminary eligibility. Inclusion criteria were: 1–18 years of age or older; 2- needed to consult a physician or referred to one of the participating clinical settings for a current knee problem; 3- able to understand and speak French. Patients previously treated by the participating physicians were excluded, as well as patients who had undergone lower limb surgery in the past six months, patients with a knee arthroplasty or who presented with more than two other lower limb pathologies or if they suffered from any systemic inflammatory disorder related to their knee complaint.

### Data collection

#### Patients’ characteristics and history elements

History elements collected included: gender, age, education level, employment status, comorbidities, affected side, duration of knee symptoms, knee pain location (anterior, posterior, medial or lateral or diffuse knee pain), traumatic or progressive onset of symptoms and use of walking aid. Possible traumatic mechanisms included: falling on knee (weight-bearing), direct external blow to the knee, trauma following a jump landing, pivoting trauma, foot/leg blocked during trauma, “popping” sensation during trauma, immediate/delayed pain, ability/inability to pursue activities and immediate/delayed effusion [22]. Participants also completed the Knee Injury and Osteoarthritis Outcome Score (KOOS), a 42-item questionnaire composed of 5 domains: pain, symptoms, function in daily living, function in sport and recreation and knee-related quality of life [27]. Psychological distress was assessed using the K6 screening scale [28].

#### Physical examination data collection procedure

Before the start of the study, clinicians met with the research personnel to standardize techniques and interpretation of the physical tests as well as the different knee diagnoses definitions. Each participant was independently assessed by two evaluators on the day of their visit: a physiotherapist and one of the participating physicians. The physiotherapist had a professional masters in physiotherapy and had one year of clinical experience. The five participating physicians (three orthopaedic surgeons and two sports medicine physicians) each had more than 20 years of experience. The physiotherapist always completed data collection prior to the physician consultation. Both the physiotherapist and the physicians were blinded to each other’s results and any other clinical information from the start of their respective evaluations. The physiotherapist did not discuss the diagnosis with the patient following his evaluation to keep the participants blinded and to ensure that their behavior at the other stage of data collection remains unchanged.

#### Physical examination tests

A complete standardized physical examination was performed by the two evaluators. Physical examination tests related to ACL tears included: Lachman, pivot shift and anterior drawer tests [11]. These tests were noted as positive/negative/uncertain/not evaluated [29, 30]. The technique for these tests and the definition of a positive response are described in Table 1.

**Table 1:** Description of physical examination tests for ACL tear.

Tests	Description of technique and positive outcomes.
Lachman [31–33]	The patient is in supine position. The clinician positions the patient’s knee at 15-30° of flexion while holding the tibial plateau with one hand and the proximal aspect of the knee with the other hand. The clinician applies a swift anterior perpendicular force to the tibia. The test is considered positive when the clinician observes an anterior glide of the tibia and/or palpates an absent end-feel significantly different from the unaffected side.
Pivot shift [31–33]	The patient is in supine position. The clinician fully extends and internally rotates the patient’s knee. The clinician’s distal hand is placed at the patient’s ankle to maintain internal rotation while his other hand palpates the lateral tibial plateau while inducing a slight valgus stress on the knee. The clinician then slowly flexes the knee. The test is considered positive if, during the first 30° of flexion, the clinician observes or palpates a subluxation and/or gliding of the tibial plateau significantly different from the unaffected side.
Anterior drawer test [31–33]	The patient is in supine position. The clinician positions the patient’s knee at 90° of flexion while stabilizing the limb by sitting on the foot of the patient. Using both hands, the clinician holds the tibial plateau and applies a slow anterior perpendicular force to the tibia. The test is considered positive when the clinician observes an anterior glide of the tibia significantly different from the unaffected side.

#### Reference standard definition

Following his independent collection of history elements and physical examination tests, the expert physician was presented with any imaging results and radiology reports and performed his own analysis of the relevant imaging tests. All participants were required to have a radiograph of their knee. Magnetic resonance imaging (MRI) was also required for all suspected ligament tears, meniscal tears or to exclude any other knee diagnoses [24]. Based on patient history, physical tests and imaging tests, the physician made a final primary and secondary (if necessary) composite diagnosis. Diagnoses were classified into 1- partial or complete ACL tear or 2- no-ACL tear. The final composite diagnosis by the physician was considered the reference standard. We compared all index tests including patients’ characteristics, history elements and physical examination tests independently collected by the physiotherapist to the composite diagnosis (reference standard) for each participant.

### Statistical analysis

Descriptive statistics were used to present the participants’ characteristics and Student t-tests as well as Chi-square tests were used to compare participants with an ACL tear to those with other knee disorders. Inter-rater reliability between the physiotherapist and the physicians was measured for all physical examination tests and diagnoses using kappa values (κ) with associated 95% CI [34]. A secondary analysis was conducted to identify only complete ACL tear cases compared to all other knee disorders including partial ACL tears, because these individuals may require a timely surgical consultation compared to those with a partial ACL tear [9, 22, 35].

Diagnostic clusters containing multiple history elements and physical examination test findings were developed using a two-step method [36]. First, Least Absolute Shrinkage and Selection Operator (LASSO) penalized logistic regression was used to identify history elements and physical examination tests predictive of the diagnosis of ACL tear either partial or complete [36, 37]. LASSO is used to select variables with a higher predictive ability in the situation where there is a large initial set of variables [36]. Variable selection by LASSO is obtained through shrinking the beta coefficients of unimportant variables to zero [38]. The degree of shrinkage is determined by a penalty parameter, the value of which is identified through cross-validation to select the set of variables that maximize area under the curve (AUC) [38].

Recursive partitioning was performed on the clinical variables selected using the LASSO to form diagnostic clusters to include and exclude a diagnosis of ACL tear, either partial or complete, and also only for complete tears [39]. Recursive partitioning allows the best sequence of variables to classify ACL tear from non-ACL tear cases [36]. The gini index was used as the splitting criteria [36, 39]. Overall model classification was compared to the reference standard (physicians’ composite diagnosis) and, sensitivity (Se), specificity (Sp), positive and negative predictive values (PPV/NPV) and positive and negative likelihood ratios (LR+/-) with 95% CI were calculated [40, 41]. Physical tests with an inter-rater agreement kappa (κ) value ≤0.4 were considered unreliable and not used in final clusters development [42]. Final selection of clusters to include and exclude ACL tear were based on overall diagnostic validity and needed to reach a LR+≥10 or a LR-<0.2. These thresholds are recognized to produce a moderate to substantial shift in post-test probability and therefore clinically useful to diagnose or exclude a disorder [43, 44]. Ease of use and clinical applicability was also considered in final selection of clusters [41]. Internal validity was assessed using a validated technique of bootstrapping for recursive partitioning and estimates with 95% CIs were compared to the proposed clusters [36, 45]. Analyses were performed using R version 3.3.0 (packages: rpart, glmnet and randomForest; http://cran.r-project.org/).

## Results

Table 2 presents the characteristics of participants. A total of 279 individuals participated (96.2% of those recruited). Participants were diagnosed with 359 primary and secondary diagnoses as follows: knee osteoarthritis (n = 129), patellofemoral pain (n = 75), symptomatic meniscal tears (n = 80) or other knee diagnoses (n = 32). Forty-three individuals (15.4%) received a diagnosis of partial (n = 21) or complete (n = 22) anterior cruciate ligament (ACL) tear. Overall, individuals with an ACL tear were significantly younger (ACL: 38.6±12.9 years old, others: 51.0±15.6 years old, p<0.05) and had a lower body mass index (ACL: 26.9±5.8, others: 29.7±6.6, p<0.05) compared to those without an ACL tear. Most individuals with an ACL tear were recruited in the orthopaedic clinics (95.3%) and had an history of trauma as the reason for consultation. (79.1%). More individuals with an ACL tear had pain for less than 3 months (ACL: 23.3%, others: 10.2%, p<0.05) and more than half were referred to surgery after consultation (ACL: 53.5%, others: 10.6%, p<0.05). For complete ACL tears only, 95.4% of individuals had a history of trauma as the reason for consultation and 68.1% were referred to surgery after consultation.

**Table 2:** Characteristics of participants (n = 279).

Characteristics	Partial or complete ACL tears (n = 43)		Other knee disorders (n = 236)	
	n (%)	mean (SD)	n (%)	Mean (SD)
Age		38.6 (12.9)*		51.0 (15.6)
Body Mass Index (Kg/m^2^)		26.9 (5.8)*		29.7 (6.6)
Sex				
*Female*	21 (48.8)		140 (59.3)	
*Male*	22 (51.2)		96 (40.7)	
Recruitment site				
*Orthopaedic clinics*	41 (95.3)*		188 (79.7)	
*Family medicine unit/university community*	2 (4.7)		48 (20.3)	
History of trauma	34 (79.1)*		53 (22.5)	
Duration of pain and symptoms at time of consultation				
*<3 months*	10 (23.3)*		24 (10.2)	
*3–12 months*	15 (34.8)*		57 (24.2)	
*≥ 12 months*	18 (41.9)*		155 (65.6)	
Referred to surgery after consultation	23 (53.5)*		25 (10.6)	
Knee Injury and Osteoarthritis Outcome Score (KOOS)				
*Pain*		67.6 (16.7)*		56.9 (20.3)
*Symptoms*		73.8 (14.5)		69.4 (19.8)
*Activity of Daily Living*		73.7 (18.3)*		64.4 (22.4)
*Sports*		28.3 (25.3)		28.7 (25.3)
*Quality of Life*		28.5 (15.6)*		40.4 (20.0)
K6 psychological distress scale (/30)		26.9 (3.5)		26.3 (4.7)

ACL: anterior cruciate ligament; SD: standard deviation; KOOS: a score of 0 indicates a severe condition and 100 indicates a normal knee; K6: a score of 6 indicates serious mental illness and 30 indicates no mental illness

* indicates a significant difference (p<0.05) between participants with an anterior cruciate ligament tear and those without.

Table 3 describes the clinical diagnoses and imaging findings for partial or complete ACL tears. Overall, ACL tear was the primary diagnosis in 63% of individuals with partial or complete ACL tears and 73% of those with complete ACL tears only. Forty-two percent of individuals with a partial or complete ACL tear and 59% with a complete tear had a combined symptomatic meniscal tear. Based on the composite reference standard diagnosis, 22 participants had a complete ACL tear, including two who had a re-rupture of a previously reconstructed ACL; 21 participants had a partially teared ACL.

**Table 3:** Description of clinical diagnoses and imaging findings for partial or complete ACL tears participants (n = 43).

	Partial or complete ACL tears (n = 43)	Partial tears only (n = 21)	Complete ACL tears only (n = 22)
Description of clinical diagnoses	n (%)	n (%)	n (%)
ACL tear primary diagnosis	27 (63)	11 (52)	16 (73)
ACL tear alone with no other knee disorder	12 (28)	5 (24)	7 (32)
ACL tear combined with another knee disorder			
*Symptomatic meniscal tear*	18 (42)	5 (24)	13 (59)
*Patellofemoral pain*	6 (14)	5 (24)	1 (5)
*Osteoarthritis*	3 (7)	2 (9)	1 (5)
*Other knee disorders*	4 (9)	4 (19)	0 (0)
**Imaging findings**			
Partial ACL tear	21 (49)	21 (100)	0 (0)
Complete ACL tear	22 (51)	0 (0)	22 (100)

ACL: anterior cruciate ligament; Clinical diagnoses are composite diagnoses made by physicians using history elements, physical examination tests and relevant imaging including magnetic resonance imaging (MRI) confirmation for all ACL tear diagnoses; Imaging findings are from MRI radiologists’ reports and/or imaging assessment by the physicians; Others knee disorders combined with ACL tears include: contusion of tibial plateau (n = 3) and lateral collateral ligament tear (n = 1).

Table 4 presents the clinical variables associated with the diagnosis of ACL tear identified through penalized logistic regression. One hundred and thirty-one different clinical variables were entered in the penalized logistic regression. Following cross-validation, six variables were associated with the diagnosis of partial or complete ACL tear and yielded a maximal area under the curve (AUC) of 0.92 (95%CI: 0.86–0.98). Four variables were history elements. A history of pivot during trauma, a popping sensation during trauma, no history of fall on the knee during trauma and no current knee pain or only monthly knee pain frequency as measured with the KOOS questionnaire were associated with a diagnosis of an ACL tear. Two physical examination tests, the Lachman and the pivot shift, were associated with the diagnosis of ACL tear. For complete ACL tears only, seven variables yielded a maximal AUC of 0.94 (95%CI: 0.91–0.97). The history element immediate effusion after trauma was associated with complete ACL tears only, while popping sensation was not. The Lachman and the pivot shift remained associated for complete ACL tears.

**Table 4:** Clinical variables associated with the diagnosis of partial or complete ACL tears at maximal AUC identified through penalized logistic regression (n = 279).

	Variables associated with a partial or complete ACL tear (n = 43)	Variables associated with a complete ACL tear only (n = 22)
**History elements**	Absence of fall on knee at initial trauma	3–4 weeks pain duration at time of consultation
	History of pivoting on knee at initial trauma	Absence of fall on knee at initial trauma
	“Popping sensation” during trauma	History of pivoting on knee at initial trauma
	No or only monthly knee pain*	Impossibility to pursue activity after trauma
		Immediate effusion after trauma
**Physical examination tests**	Lachman	Lachman
	Pivot shift	Pivot shift

ACL: anterior cruciate ligament; LASSO: Least Absolute Shrinkage and Selection Operator; Maximal area under the curve (AUC) was used as the criteria for the final penalty parameter to select variables associated with the presence or absence of ACL tear. Complete ACL tears only was compared to all disorders, including partial tears.

*KOOS question for pain frequency: How often do you experience knee pain: never, monthly, weekly, daily, always. Timing of effusion after trauma included: no effusion, immediate effusion, effusion appeared more than 2 hours after trauma. Pain duration at time of consultation included: 1–2 weeks, 3–4 weeks, 5–6 weeks, 7–8 weeks, 9–11 weeks, 3–6 months, 6–9 months, 9–12 months, more than 12 months.

Table 5 presents the diagnostic validity and reliability of history elements and physical examination tests when individually performed. The Lachman reached inter-rater kappa (κ) value of 0.75 (95%CI: 0.63–0.88). The pivot shift reached inter-rater kappa value of 0.84 (95%CI: 0.65–1.00).

**Table 5:** Diagnostic validity and inter-rater reliability of history elements and physical examination tests when individually performed to diagnose or exclude partial or complete ACL tears (n = 43) or complete ACL tears only (n = 22).

	Se (95% CI)		Sp (95% CI)		LR- (95% CI)		LR+ (95% CI)		Inter-rater reliability (95% CI)
	Partial or complete	Complete only	Partial or complete	Complete only	Partial or complete	Complete only	Partial or complete	Complete only	
**History elements**									
Pivoting traumatic mechanism	0.79 (0.64–0.90)	0.95 (0.77–1.00)	0.84 (0.79–0.89)	0.81 (0.75–0.85)	0.25 (0.14–0.44)	0.06 (0.01–0.38)	5.0 (3.6–7.0)	4.9 (3.8–6.4)	-
“Popping” sensation	0.58 (0.42–0.73)	-	0.92 (0.88–0.95)	-	0.45 (0.32–0.65)	-	7.6 (4.6–12.7)	-	-
Immediate effusion after trauma	-	0.68 (0.45–0.86)	-	0.94 (0.90–0.96)	-	0.34 (0.18–0.63)	-	11.0 (6.3–19.1)	-
**Physical examination tests**									
Lachman	0.81 (0.67–0.92)	0.82 (0.60–0.95)	0.98 (0.95–0.99)	0.91 (0.87–0.95)	0.19 (0.10–0.36)	0.20 (0.08–0.48)	38.4 (16.0–92.5)	9.6 (6.1–14.9)	0.75 (0.63–0.88)
Pivot shift	0.77 (0.61–0.89)	0.80 (0.56–0.94)	0.98 (0.95–0.99)	0.92 (0.87–0.95)	0.24 (0.13–0.42)	0.22 (0.09–0.53)	37.5 (14.0–100.4)	9.5 (5.8–15.6)	0.84 (0.65–1.00)

ACL: anterior cruciate ligament; Se: sensitivity; Sp: specificity; LR+: positive likelihood ratio; LR-: negative likelihood ratio. “Popping” sensation was not calculated for complete ACL tears and immediate effusion was not calculated for partial and complete tears because it did not show an association with LASSO results presented in Table 4. For Lachman, cases coded as “Uncertain” were considered “Positive” for a suspicion of ACL tear (n = 3). Inter-rater reliability (kappa) was measured only for physical examination tests. The pivot shift could not be evaluated in n = 45 patients (n = 2 partial ACL tears, n = 2 complete ACL tears, 41 non-ACL tears).

A positive “popping” sensation presented a LR+ of 7.6 (95%CI: 4.6–12.6) to diagnose both partial and complete ACL tears. A negative pivoting traumatic mechanism had a LR- of 0.25 (95%CI: 0.14–0.44) to exclude partial or complete ACL tears. The Lachman test reached LR+ of 38.4 (95%CI: 16.0–92.5) and LR- of 0.19 (95%CI: 0.10–0.36) to diagnose and exclude partial or complete ACL tears. The pivot shift reached a LR+ of 37.5 (95%CI: 14.0–100.4) and LR- of 0.24 (95%CI: 0.13–0.42) to diagnose and exclude partial or complete ACL tears.

Immediate effusion after trauma presented a LR+ of 11.0 (6.3–19.1) to diagnose a complete ACL tear and a negative pivoting traumatic mechanism reached a LR- of 0.06 (95%CI: 0.01–0.38) to exclude a complete ACL tear. The Lachman test presented a LR+ of 9.6 (95%CI: 6.1–14.9) and LR- of 0.20 (0.08–0.48) to diagnose and exclude complete ACL tears compared to all knee disorders including partial ACL tears. The pivot shift reached LR+ of 9.5 (95%CI: 5.8–15.6) and LR- of 0.22 (0.09–0.53) to diagnose and exclude complete ACL tears compared to all knee disorders including partial ACL tears.

Diagnostic clusters combining history elements and physical examination tests were identified through recursive partitioning using the clinical variables associated with a diagnosis of ACL tears. Table 6 presents two diagnostic clusters to diagnose partial or complete ACL tears or complete ACL tears only compared to other knee disorders. One cluster combined only two history elements: pivoting traumatic mechanism with a “popping” sensation during trauma to diagnose partial or complete ACL tears. The cluster correctly classifies 25/43 individuals with an ACL tear (see 2x2 table in S1 Tables) and has the following diagnostic statistics: Se of 0.58 (95%CI: 0.42–0.73), Sp of 0.94 (95%CI: 0.90–0.97, PPV of 0.64 (95%CI: 0.47–0.79), LR+ of 9.80 (95%CI: 5.55–17.29).

**Table 6:** Diagnostic clusters combining history elements and physical examination tests to diagnose partial or complete ACL tears (n = 43) or complete ACL tears only (n = 22).

Clusters	Se (95% CI)	Sp (95% CI)	PPV (95% CI)	LR+ (95% CI)
**Knee complaints are likely due to a partial or complete ACL tear in individuals with:**	0.58 (0.42–0.72)	0.94 (0.90–0.97)	0.64 (0.47–0.79)	9.80 (5.55–17.29)
➢Pivoting traumatic mechanism				
*AND*				
➢“Popping” sensation during trauma				
Internal validation	0.53 (0.38–0.69)	0.92 (0.87–0.95)	0.56 (0.40–0.72)	7.01 (4.15–11.85)
**Knee complaints are likely due to a complete ACL tear in individuals with:**	0.82 (0.60–0.95)	0.95 (0.92–0.97)	0.60 (0.41–0.77)	17.5 (9.8–31.5)
➢Pivoting traumatic mechanism				
*AND*				
➢Immediate effusion after trauma				
*AND*				
➢Positive Lachman test				
Internal validation	0.82 (0.60–0.95)	0.93 (0.90–0.96)	0.51 (0.34–0.69)	12.37 (7.50–20.39)

Se: sensitivity; Sp: specificity; PPV: positive predictive value; LR+: positive likelihood ratio. Clusters are obtained using recursive partitioning with all variables associated with the diagnosis of an ACL tear. Internal validation was assessed by bootstrapping (n = 1000).

The other cluster (Table 6) combined a pivoting traumatic mechanism with immediate effusion after trauma and a positive Lachman test to diagnose complete ACL tears only compared to other disorders (including partial tears). The cluster correctly identifies 18/22 of cases (see 2x2 table in S1 Tables) and has the following diagnostic statistics: Sp of 0.95 (95%CI: 0.92–0.98), PPV of 0.60 (95%CI; 0.41–0.77) and LR+ of 17.5 (95%CI: 9.8–31.5).

Table 7 presents one diagnostic cluster to exclude partial or complete ACL tears compared to other knee disorders. The cluster combined a negative history of pivot or popping sensation during trauma with a negative Lachman or pivot shift test to exclude an ACL tear. The cluster correctly classifies 206/236 individuals without a partial or complete ACL tear (see 2x2 table in S1 Tables). The cluster shows the following diagnostic statistics: Se of 0.93 (95%CI: 0.81–0.99), Sp of 0.87 (95%CI: 0.82–0.91, NPV of 0.99 (95%CI: 0.96–1.00), LR- of 0.08 (95%CI: 0.03–0.24). Tables 6 and 7 also present internal validation estimates and 95% CIs obtained using bootstrapping (n = 1000).

**Table 7:** Diagnostic cluster combining history elements and physical examination tests to exclude partial or complete ACL tears (n = 43).

Clusters	Se (95% CI)	Sp (95% CI)	NPV (95% CI)	LR- (95% CI)
**Knee complaints are** **unlikely due** **to a partial or complete ACL tear in individuals with:**	0.93 (0.81–0.99)	0.87 (0.82–0.91)	0.99 (0.96–1.00)	0.08 (0.03–0.24)
➢ Negative history of pivot or “popping” sensation during trauma				
*AND*				
➢ Negative Lachman or pivot shift test				
Internal validation	0.91 (0.78–0.97)	0.85 (0.80–0.89)	0.98 (0.95–0.99)	0.11 (0.04–0.27)

Se: sensitivity; Sp: specificity; NPV: negative predictive value; LR-: negative likelihood ratio. Clusters are obtained using recursive partitioning with all variables associated with the diagnosis of an ACL tear. Internal validation was assessed by bootstrapping (n = 1000).

## Discussion

This study assessed the diagnostic validity of clusters combining history elements and physical examination tests to diagnose or exclude partial or complete ACL tears compared to other knee disorders. We first observed that when individually performed, the Lachman and the pivot shift tests reached substantial positive LR (LR+>10) to diagnose and moderate negative LR (LR- <0.2) to exclude partial or complete ACL tears [44]. In the present cohort, this increased positive post-test probability by 72.6% and negative post-test probability by 12.4% and 10.4% for both tests. These results are comparable with data synthesized in a previous meta-analysis reporting substantial positive LR to diagnose a partial or complete ACL tear using these tests (Lachman: LR+ = 10.2 (95%CI: 4.6–22.7), pivot shift: LR+ = 12.0 (95%CI: 5.0–31.0)) and moderate negative LR to exclude an ACL tear (Lachman: LR- = 0.2 (95%CI: 0.10–0.30)) [16]. Taken alone these tests can be considered clinically useful to diagnose or exclude a partial or complete ACL tear.

We also demonstrated that combining history elements and physical examination tests improved the probability to diagnose or exclude partial or complete ACL tears. The first cluster, combining only history elements, presented near substantial positive LR (LR+ = 9.80) [44], increasing positive post-test probability by 48.6% for partial or complete ACL tears. A second cluster combining two history elements and adding a positive Lachman test demonstrated a substantial positive LR to diagnose complete ACL tears only, increasing positive post-test probability by 52.1%. The last cluster combined a negative history of pivot or popping sensation during trauma with a negative Lachman or pivot shift tests, and was able to exclude both partial or complete ACL tears (LR-<0.1) [44]. Combining only history elements accurately identified 58% of all ACL tears cases in this cohort, while combining history elements and physical examination tests accurately identified more than 80% of all cases and non-cases.

Our study compares favorably with previous evidence on the combination of selected history elements and physical examination tests. In a sample of 134 acute traumatic patients including 28 with a partial or complete ACL tear, *Wagemaker et al*. found that combining immediate effusion, popping sensation, giving way symptoms and a positive anterior drawer test yielded a positive LR of 4.2 for partial or complete tears (95%CI: 2.4–7.5) and a positive LR of 7.2 for complete tears only (95%CI: 3.6–14.4) [22]. In another study by *Geraets et al*., with a sample of 60 patients undergoing knee arthroscopy and including 22 complete ACL tears, the authors found that combining immediate effusion, popping sensation and complaints of giving way as well as positive Lachman, pivot shift and anterior drawer tests yielded a positive LR of 24.0 [23]. Although somewhat comparable, the differences observed in the magnitude of the estimates between our study and these two studies may be explained by several factors. The inclusion of more acute patients in the first study may have made the physical examination tests more difficult to perform and interpret because of knee effusion or muscle guarding [22, 35]. In our study, most participants (77%) had their trauma more than 3 months before their evaluation and this may have made it easier to perform the physical tests and interpret these tests, ultimately increasing diagnostic validity estimates. Nevertheless, combining only history elements, which are not affected by an acute presentation, also showed accurate validity in our study to support the diagnosis of partial or complete ACL tears.

Another factor explaining the difference between our study and the literature may be related to the representativeness of included participants. In both studies by *Wagemaker et al*. and *Geraets et al*., the non-ACL tear patients included only participants with a traumatic history which may limit the external validity of their findings [22, 23]. In our study, we compared patients with a partial or complete ACL tear to other patients with various knee disorders, not only suffering from a traumatic onset. This allowed us to demonstrate that the traumatic onset of symptoms was a diagnostically useful history element when discriminating between several knee disorders. The fact that cases and non-cases were more chronic in our study and not all of them had a history of trauma could have inflated the diagnostic capability of our results, yet it may at the same time limit spectrum bias commonly seen in diagnostic studies in this field [11, 22, 23, 46]. The external validity of our findings will need to be assessed in a validation study with another cohort of participants.

Previously published diagnostic studies reported the inclusion of the anterior drawer test in their diagnostic models [22, 23]. In our cohort, we did not found an association between a positive anterior drawer test and the diagnosis of ACL tear. This may be because our cohort includes patients with both partial or complete ACL tears combined with meniscal tears which have been shown to lower the diagnostic validity of this test [11, 15, 16, 35, 47]. Based on our results, the anterior drawer test produced twice as much false-negative cases as the Lachman test (data not shown). Even if this test may appear easier to learn by untrained clinicians, the false-negative rate precluded us from proposing the anterior drawer test as a valid test at this time [22]. Moreover, even if the Lachman and pivot shift tests are often found to be harder to perform and interpret, we showed higher inter-rater reliability for both tests than in previous reliability results [29, 48]. A likely explanation may be that we used a dichotomous outcome (positive/negative) as originally described in the initial publication of these tests [30]. Also, evaluators assessed both knees and comparison to the unaffected side may have supported higher confidence in a positive or negative response to the tests, hence higher inter-rater reliability.

### Strengths and limitations

We recruited a prospective cohort from different settings allowing for a broad variety of cases and non-cases. However, the main limitation of this study is that most individuals with an ACL tear were found in secondary care and the performance of the clusters may be different in primary care patients with an acute presentation (e.g.: family medicine clinics, emergency departments, physiotherapy clinics). Thus, our findings are not completely representative of all patients from the general population consulting for an ACL tear.

Our data collection procedure ensured blinding of the evaluators between the index tests and the reference standard. However, even though we included only new patients to ensure blinding, most of our ACL tear patients were exposed to other physicians prior to obtaining an appointment with the expert physicians and this could have influenced their responses to history and physical examination tests. Another limitation of our data collection procedure was that the physiotherapist, even if blinded to the diagnosis, collected history elements prior to obtaining the physical examination tests to replicate the usual diagnostic process, but this may have influenced his interpretation of these tests. Also, results from the physical examination tests were only dichotomized (positive or negative) and we did not take into account the end point feeling or the amount of anterior translation or subluxation of the tibia, often assessed by clinicians when performing these laxity tests.

Our composite reference standard by expert physicians included history elements, physical examination tests as well as MRI confirmation for all ACL tears. A composite reference standard is advocated because meta-analyses showed that use of MRI only as the reference standard reached only a Se of 86.5% and a Sp of 95.2% [49–52]. However, only one expert physician assessed each patient to obtain the reference standard. A previous analysis demonstrated that the diagnostic concordance between the physiotherapist and the physician was very high [53]. Lastly, our statistical approach (LASSO and recursive partitioning) lead to the identification of simple and clinically useful clusters based on an extensive set of patient’s personal and clinical variables [36].

## Conclusions

We identified diagnostic clusters combining history elements and physical examination tests that can accurately support the differential diagnosis of partial or complete ACL tears compared to other knee disorders. Given adequate external validation and appropriate clinical training by clinicians, these clusters could be used by health providers involved in musculoskeletal care in settings with limited access to MRI and initiate an accelerated rehabilitation protocol or propose a specialty referral when surgery is considered.

## Supporting information

S1 Fig (DOCX)

S1 Highlights (DOCX)

S1 Tables (DOCX)
