# Supplementary material for: Clinical diagnosis of partial or complete anterior cruciate ligament tears using patients' history elements and physical examination tests
Source: PLoS One. 2018 Jun 12;13(6):e0198797. doi: 10.1371/journal.pone.0198797 (PMC5997333; doi:10.1371/journal.pone.0198797)
Supplement: S1 Fig — (DOCX) [file pone.0198797.s001.docx]

**Figure 1: Flow chart of patient recruitment**

Patients approached to participate or who volunteered

n=307

Refused to participate

n=11

*Lack of time (n=5)*

*Not interested (n=6)*

Patients included

n=279

Excluded before consultation

n=17

*Total knee arthroplasty (n=1)*

*Prior visit with one of the participating physicians (n=4)*

*No knee complaint (n=1)*

*Systemic rheumatoid*

*disorders (n=3)*

*Under 18 years old (n=1)*

*Did not understand French (n=4)*

*Not able to consent or mental health disorders (n=3)*
