# Supplementary material for: Clinical diagnosis of partial or complete anterior cruciate ligament tears using patients' history elements and physical examination tests
Source: PLoS One. 2018 Jun 12;13(6):e0198797. doi: 10.1371/journal.pone.0198797 (PMC5997333; doi:10.1371/journal.pone.0198797)
Supplement: S1 Highlights — (DOCX) [file pone.0198797.s002.docx]

**Highlights**

- The Lachman physical examination test is valid to support the diagnosis of a partial or complete ACL tear when individually performed.
- The combination of a pivoting traumatic mechanism with a “popping” sensation during trauma can support the diagnosis of a partial or complete ACL tear.
- Combining a negative history of pivot or a negative popping sensation during trauma with a negative Lachman or pivot shift test is valid to exclude a partial or complete ACL tear.
- The Lachman and pivot shift tests reached high inter-rater reliability between a physiotherapist and expert physicians.
