# Supplementary material for: Clinical diagnosis of partial or complete anterior cruciate ligament tears using patients' history elements and physical examination tests
Source: PLoS One. 2018 Jun 12;13(6):e0198797. doi: 10.1371/journal.pone.0198797 (PMC5997333; doi:10.1371/journal.pone.0198797)
Supplement: S1 Tables — (DOCX) [file pone.0198797.s003.docx]

**Table 1: 2x2 tables of diagnostic validity for history elements and physical examination tests when individually performed for partial or complete ACL tears**

| **Pivoting traumatic mechanism** | | | | |
| --- | --- | --- | --- | --- |
|  | | Reference standard | | |
|  |  | Yes | No | Total |
| Classification | Yes | 34 | 37 | 71 |
|  | No | 9 | 199 | 208 |
|  | Total | 43 | 236 | 279 |

| **Popping sensation during trauma** | | | | |
| --- | --- | --- | --- | --- |
|  | | Reference standard | | |
|  |  | Yes | No | Total |
| Classification | Yes | 25 | 18 | 43 |
|  | No | 18 | 218 | 236 |
|  | Total | 43 | 236 | 279 |

| **Lachman** | | | | |
| --- | --- | --- | --- | --- |
|  | | Reference standard | | |
|  |  | Yes | No | Total |
| Classification | Yes | 35 | 5 | 40 |
|  | No | 8 | 231 | 239 |
|  | Total | 43 | 236 | 279 |

| **Pivot Shift** | | | | |
| --- | --- | --- | --- | --- |
|  | | Reference standard | | |
|  |  | Yes | No | Total |
| Classification | Yes | 30 | 4 | 34 |
|  | No | 9 | 191 | 200 |
|  | Total | 39 | 195 | 234 |

**Table 2: 2x2 tables of diagnostic validity for history elements and physical examination tests when individually performed for complete ACL tears only**

| **Pivoting traumatic mechanism** | | | | |
| --- | --- | --- | --- | --- |
|  | | Reference standard | | |
|  |  | Yes | No | Total |
| Classification | Yes | 21 | 50 | 71 |
|  | No | 1 | 207 | 208 |
|  | Total | 22 | 257 | 279 |

| **Immediate effusion** | | | | |
| --- | --- | --- | --- | --- |
|  | | Reference standard | | |
|  |  | Yes | No | Total |
| Classification | Yes | 15 | 16 | 31 |
|  | No | 7 | 241 | 248 |
|  | Total | 22 | 257 | 279 |

| **Lachman** | | | | |
| --- | --- | --- | --- | --- |
|  | | Reference standard | | |
|  |  | Yes | No | Total |
| Classification | Yes | 18 | 22 | 40 |
|  | No | 4 | 235 | 239 |
|  | Total | 22 | 257 | 279 |

| **Pivot Shift** | | | | |
| --- | --- | --- | --- | --- |
|  | | Reference standard | | |
|  |  | Yes | No | Total |
| Classification | Yes | 16 | 18 | 34 |
|  | No | 4 | 196 | 200 |
|  | Total | 20 | 214 | 234 |

**Table 3: 2x2 tables of diagnostic clusters using recursive partitioning for partial or complete ACL tears**

| **Combination of history with pivot and popping sensation during trauma** | | | | |
| --- | --- | --- | --- | --- |
|  | | Reference standard | | |
|  |  | Yes | No | Total |
| Classification | Yes | 25 | 14 | 39 |
|  | No | 18 | 222 | 240 |
|  | Total | 43 | 236 | 279 |

| **Combination of negative history with pivot or popping sensation during trauma and negative Lachman or pivot shift** | | | | |
| --- | --- | --- | --- | --- |
|  | | Reference standard | | |
|  |  | Yes | No | Total |
| Classification | Yes | 40 | 30 | 70 |
|  | No | 3 | 206 | 209 |
|  | Total | 43 | 236 | 279 |

**Table 4: 2x2 tables of diagnostic clusters using recursive partitioning for complete ACL tears only**

| **Combination of history of pivot during trauma and immediate effusion after trauma and a positive Lachman test** | | | | |
| --- | --- | --- | --- | --- |
|  | | Reference standard | | |
|  |  | Yes | No | Total |
| Classification | Yes | 18 | 12 | 30 |
|  | No | 4 | 245 | 249 |
|  | Total | 22 | 257 | 279 |
